# Chromosome Painting in Three Species of Buteoninae: A Cytogenetic Signature Reinforces the Monophyly of South American Species

**DOI:** 10.1371/journal.pone.0070071

**Published:** 2013-07-26

**Authors:** Edivaldo Herculano C. de Oliveira, Marcella Mergulhão Tagliarini, Michelly S. dos Santos, Patricia C. M. O'Brien, Malcolm A. Ferguson-Smith

**Affiliations:** 1 Laboratório de Cultura de Tecidos e Citogenética, SAMAM, Instituto Evandro Chagas, Ananindeua, PA, Brazil; 2 Faculdade de Ciências Exatas e Naturais, ICEN, Universidade Federal do Pará, Belém, PA, Brazil; 3 Cambridge Resource Centre for Comparative Genomics, Cambridge, United Kingdom; 4 Programa de Pós Graduação em Neurociências e Biologia Celular, ICB, Universidade Federal do Pará, Belém, PA, Brazil; 5 PIBIC – Universidade Federal do Pará, Belém, PA, Brazil; University of Florence, Italy

## Abstract

Buteoninae (Falconiformes, Accipitridae) consist of the widely distributed genus Buteo, and several closely related species in a group called “sub-buteonine hawks”, such as Buteogallus, Parabuteo, Asturina, Leucopternis and Busarellus, with unsolved phylogenetic relationships. Diploid number ranges between 2n = 66 and 2n = 68. Only one species, L. albicollis had its karyotype analyzed by molecular cytogenetics. The aim of this study was to present chromosomal analysis of three species of Buteoninae: Rupornis magnirostris, Asturina nitida and Buteogallus meridionallis using fluorescence in situ hybridization (FISH) experiments with telomeric and rDNA probes, as well as whole chromosome probes derived from Gallus gallus and Leucopternis albicollis. The three species analyzed herein showed similar karyotypes, with 2n = 68. Telomeric probes showed some interstitial telomeric sequences, which could be resulted by fusion processes occurred in the chromosomal evolution of the group, including the one found in the tassociation GGA1p/GGA6. In fact, this association was observed in all the three species analyzed in this paper, and also in L. albicollis, suggesting that it represents a cytogenetic signature which reinforces the monophyly of Neotropical buteoninae species.

## Introduction

The study of genome organization and participation of chromosome rearrangements in speciation and macroevolutionary events is fundamental to understanding the dynamics of chromosomes [1]. Despite this, structure and role of nucleic acids and proteins have usually been the only or main focus of evolutionary researches, and chromosomes and homologous synteny blocks have been ignored [2]. Hence, chromosomal data remain underutilized in phylogenetic investigations, especially in groups such as birds, in which comparative studies have fueled advances in cytotaxonomy only after molecular cytogenetics approaches. One groups with a higher number of species analyzed by cytogenetics means is the family Accipitridae (Aves, Falconiformes) is the fourth largest non-passerine family (approximately 240 species) comprised of diurnal birds of prey or raptors (hawks, eagles, vultures), one third of which occur in the Neotropics [3]–[5]. Compared to most birds, the karyotype of accipitrides shows a different organization, with lower diploid numbers and few pairs of microchromosomes [3].

Among the classic groups of accipitrids are the buteonine hawks, which consist of the widely distributed genus *Buteo*, with 28 species occuring on all continents except Antarctica and Australia [5], and several closely related species in a group called “sub-buteonine hawks”, such as *Buteogallus, Parabuteo, Asturina, Leucopternis* and *Busarellus*, among others. This group is defined by some authors as a subfamily, Buteoninae [6], [7], but formal sub-familial division of Accipitridae has been a contentious issue due to a lack of knowledge on the evolutionary history of the family [8]. The Buteoninae are of particular interest, because 11 species are of conservation concern, with one critically endangered species (*Buteo ridgwayi*) and two endangered species (*Leucopternis occidentalis* and *Harpyhaliaetus coronatus*) [9].

Phylogenetic relationships among the species of this group remain unsolved. Molecular studies have brought some resolution to this issue [10]–[15]. In one of the most complete studies including 54 Neotropical species of Buteoninae, a phylogeny based on mitochondrial markers highlighted previous discoveries of paraphyly in three genera (*Buteo, Butelogallus* and *Leucopternis*) [12]. According to the authors, this paraphyly establishes a lack of concordance between present Accipitridae taxonomy and mtDNA phylogeny for the group, and reinforces the need for further analysis including different methodologies in all taxonomic levels in the family.

Chromosomal analyses of Buteoninae species showed that they share the same characteristics of other accipitrid bird lineages, in which in most instances, the chromosomal repatternings are centric fusions and translocations, which displace telomeric sites, originate new associations of syntenic blocks, and cause a decrease in the diploid numbers. Classical cytogenetic data showed that diploid numbers vary from 54 to 82 chromosomes in Accipitridae; however, species with 2n = 66–68 are in the majority [3], [16]. Except for *Leucopternis albicollis,* with 2n = 66 [16], all other species of Buteoninae have 2n = 68 [17]. Because accipitrids have the most ‘atypical organization’ known in the class Aves, with reduction of microchromosomes (4–6 pairs) and relatively low diploid numbers, many species of this group have been analyzed by means of classical cytogenetics. On the other hand, chromosome painting data with chicken probes are available for five species of Accipitridae: the Harpy eagle (*Harpia harpija*) [18], three species of Old World vultures (*Gyps rupelli, Gyps fulvus* and *Gypaetus barbatus*) [19], and one species of Neotropical buteoninae, *Leucopternis albicollis* [17]. These studies confirmed the occurrence of fusions and translocations involving microchromosomes which reduced their number, while multiple fissions and fusions increased the number of biarmed chromosomes.

*Leucopternis albicollis* has also been used to generate whole-chromosome painting probes. The karyotype of this species was found to be highly derived when compared to the typical avian complement, and cross-species hybridization between LAL and GGA confirmed the occurrence of fissions in some chicken macrochromosomes, and fusions involving segments homologous to chicken microchromosomes and macrochromosomes. For example, GGA 1 was found reorganized in five different pairs (LLA 3, 7, 6, 15 and 18). In addition, LAL 3 is homologous to an association of GGA1p/GGA6, with a remaining interstitial telomeric sequence [17]. Despite the number of species of Accipitridae analyzed by ZOO-FISH, there is still a lack of information concerning the cytogenomics of this group. Hence, the aim of this study was to present chromosomal analysis of three species of Buteoninae: *Rupornis magnirostris, Asturina nitida* and *Buteogallus meridionallis* using molecular cytogenetic data obtained by fluorescence *in situ* hybridization (FISH) with rDNA probes and whole chromosome probes derived from *Gallus gallus* and *Leucopternis albicollis*. The results add to our understanding of phylogenetic relationships among these Neotropical buteonines.

## Materials and Methods

### Cell Samples and Metaphase Chromosome Preparation

Experiments followed ethical protocols. The process was approved by the CNPq committee under no. 300818/2009-7. and collecting permit was obtained from SISBIO under no. 34199-1. Feather pulps were obtained from captive individuals maintained in Zoos: a female of *Rupornis magnirostris* (RMA), a male and a female of *Asturina nitida* (ANI), and a male and a female of *Buteogallus meridionallis* (BME). Cell cultures were performed according to Sasaki *et al.* [20] with modifications, initiated using dissociated cells following incubation in collagenase for 1 h. Chromosomes were obtained by standard arrest with colcemid (Gibco), hypotonic treatment with 0.075 M KCl, and cell fixation in methanol/acetic acid (3∶1). Diploid number definition and karyotype ordering were performed in conventionally stained metaphases (Giemsa 5% in 0.07 M phosphate buffer, pH 6.8).

### In situ Hybridization and Probe Detection

After treatment with pepsin (3 minutes in 0,01% pepsin solution), slides were dehydrated in ethanol (70%, 90%, and 100%) and incubated at 37°C overnight. *In situ* hybridization using biotin labeled 18S/28S human ribosomal DNA probes and signal detection were carried out using standard techniques as described earlier [21]. Experiments using whole chromosome probes from *Leucopternis albicollis* (LAL) (LAL 1 to LAL 20) and *Gallus gallus* (GGA1 to GGA13 and Z) were performed according to de Oliveira *et al* [17]. Chromosomes were counterstained with DAPI. Hybridization results were examined and analyzed using a Zeiss Imager 2 fluorescent microscope and Axiovision 4.8 software (Zeiss, Germany).

## Results

**Figure 1:**
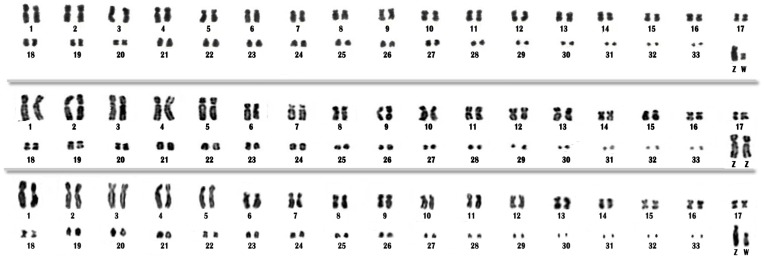
Conventional karyotype of *Asturina nitida* (A), *Rupornis magnirostris* (B) and *Buteogallus meridionalis* (C).

Good quality chromosome suspensions were obtained from the three species here analyzed. 18S rDNA and whole-chromosome specific probes delivered reproducible results when hybridized to metaphases of *Rupornis magnirostris, Buteogallus meridionallis* and *Asturina nitida.* The three species analyzed herein showed similar karyotypes, with 2n = 68, with slight differences in chromosome morphology affecting the length of arms (Fig. 1). The first 19 pairs (*R. magnirostris* and *Buteogallus meridionallis*) or 18 pairs (*A. nitida*) were biarmed chromosomes (except pair 6, acrocentric), while the remaining were acrocentric elements. We considered pairs 28–33 as microchromosomes, for their smaller size when compared to the others. The Z chromosome was a larger submetacentric element, while the W was metacentric in *R. magnirostris* and submetacentric in *A. nitida*.

**Figure 2:**
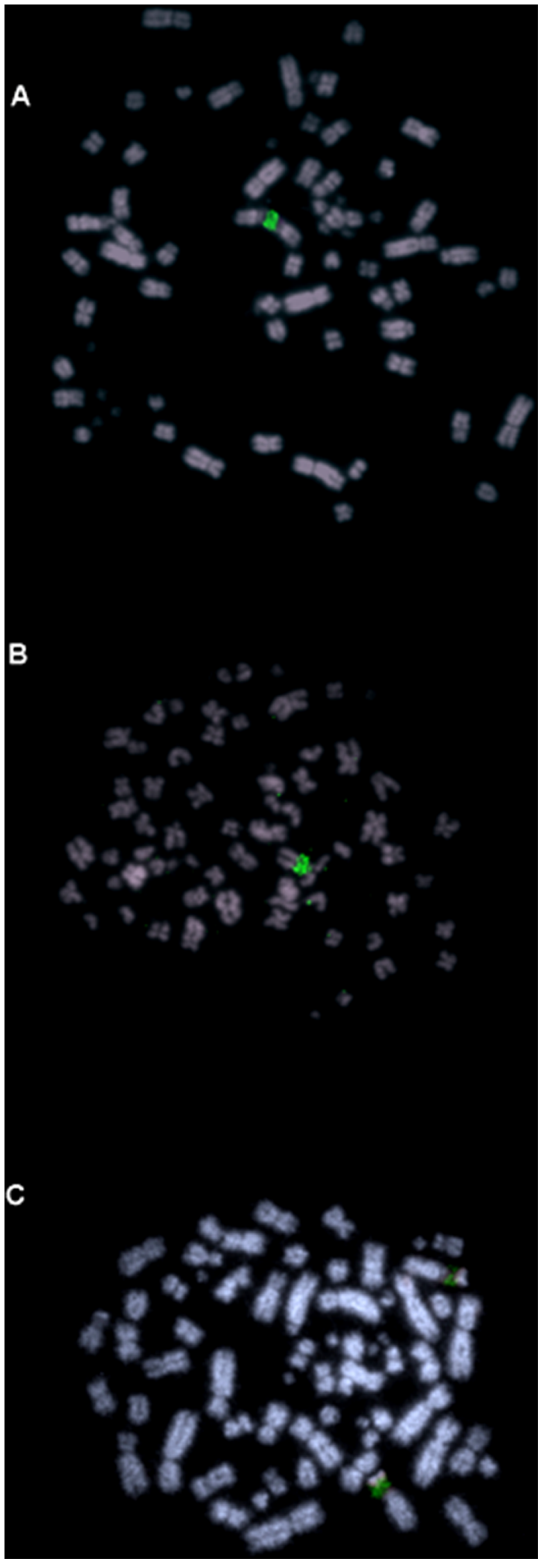
18S-45SrDNA probes (green) and telomeric probes (red) in *Asturina nitida* (A and B), *Rupornis magnirostris* (C and D) and *Buteogallus meridionallis* (E and F). Note that in (A) and (C), NOR-bearing chromosomes are associated. Arrows show interstitial telomeric sequences.

FISH experiments using 18S/28S rDNA gene fragments as probes mapped major ribosomal gene clusters in the short arm of pair 7(Fig. 2 a–c), in which it is possible to visualize a secondary constriction by conventional staining in some metaphases. For each individual at least 20 complete metaphase plates were studied.

**Figure 3:**
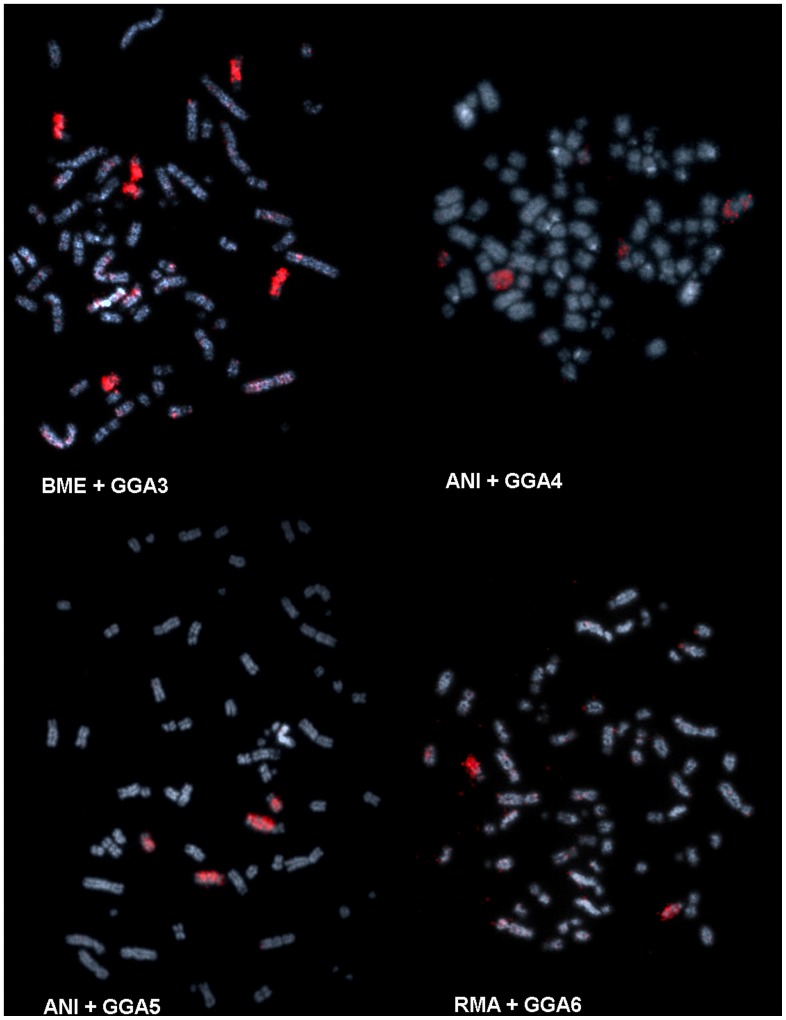
Representative FISH experiments using whole chromosome probes derived from Gallus (GGA) onto *Buteogallus meridionallis* (BME), *Asturina nitida* (ANI) and Rupornis *magnirostris* (RMA).

**Figure 4:**
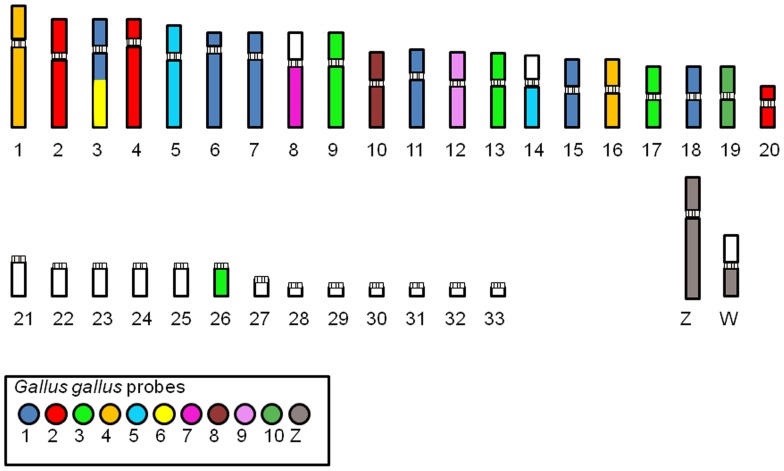
Homology map between Buteoninae karyotype and chicken paints. Correspondences are indicated by colors. White sections represent segments not hybridized by any of the applied probes.

Whole chromosome probes of *Gallus gallus* comprising the first three pairs (GGA1-3) hybridized onto multiple pairs in *R. magnirostris, B. meridionallis* and *A. nitida*: GGA1 correponded to 4 different pairs, and also onto part of pair 3 (3p and 3qprox), GGA2 to 3, and GGA3 to 4 different pairs. GGA4 and GGA5 hybridized onto a large biarmed pair (1 and 5, respectively) and to a small pair each; GGA6 onto two thirds of 3q and GGA7 onto the long arm of pair 8. The other probes (GGA8-GGA13) hybridized onto one pair each. GGA Z produced signals not only on the Z chromosome, but also on the long arm of the W chromosome. Whole chromosome probes derived from *Leucopternis albicollis* produced signals in one pair of chromosomes of *R. magnirostris*, *B. meridionallis* and *A, nitida* each. Representative results of FISH experiments are shown in figure 3, and a homology map is shown in figure 4.

## Discussion

Around 70 species of Falconiformes have been the subject of cytogenetic analysis [2], [15]–[19], corresponding to more than 20% of the total number of species in this order. Although it seems a low percentage, birds of prey are among the groups with the largest number of species with some karyological data. Buteoninae species have shown a diploid number of 66 or 68 chromosomes [2], [16]–[17]. A possible explanation for this difference is a fusion between two small sized chromosomes in *L. albicollis* (2n = 66), considering that each whole chromosome probe of the largest chromosomes from this species hybridized onto only one pair of *A. nitida, R. magnirostris* and *B. meridionallis* (2n = 68).

Analysis of the karyotypes of ten different species of Buteoninae by Schmutz *et al* [16] led the authors to divide them into three groups based on the morphology of the chromosomes, with 15, 19 or 20 biarmed autosomal pairs. However, *A. nitida* and *R. magnirostris* were included in the group with 19 biarmed chromosomes, while in our results these species showed 20. Agreeing with our results, these authors found one pair of NOR bearing chromosomes. Chromosome painting showed that this pair corresponds to an association between GGA 7 and a microchromosome, since GG7 hybridized onto the whole long arm of this pair, and no other macrochromosome probe has produced any signals in its short arm. The location of NORs associated with GGA7 is also different from the result of Harpy eagle, in which NORs were observed in the short arm of pair 8 (an association between GGA6 and a microchromosome) [18]. There is no data concerning NOR positions in other Accipitridae species using FISH analyses.

Whole chromosome probes derived from GGA1-10 produced the same number of signals in the three species, corresponding to the results found in *L. albicollis* [17]. As a control, probes derived from *L. albicollis* hybridized onto one chromosome pair each, confirming that no interchromosomal rearrangements involving the largest pairs have occurred in the karyotype of these species. Hence, it can be argued that the differences found in the number of biarmed chromosomes were resulted from intrachromosomal rearrangements (pericentric inversion or centromeric shift) or fusions involving two microchromosomes in the case of *L. albicollis*. The comparison with other species of Accipitridae, as well as with Falconidae (table 1), shows a general tendency of fragmentation of macrochromosomes of *Gallus* (supposedly similar to syntenic groups found in the putative ancestral karyotype (PAK) for birds, except for GGA4, which correspond to two different elements – pairs 4 and 10) [14]. It is also notable that syntenic groups correspond to GGA4p/GGA4q, GGA6, GGA7, GGA8, GGA9 and GGA10 tend to be conserved, although sometimes fused with other elements, but never fragmented in birds of prey. Moreover, a clear dichotomy is observed when comparing *Falco* species (Falconidae) and Accipitridae species, concerning the reshuffling of GGA1-3, which have split up into two elements in *Falco*, while in Accipitridae this number ranges from 3–6 derived chromosome pairs [17]–[19], [22].This fact can, in part, explain the lower diploid numbers observed in *Falco*, with 2n = 40–52 [22]. Moreover, an important area to be investigated is the centromeric/telomeric sequence dynamics in the process of chromosomal evolution in this group.

**Table 1:** Homoeologous chromosomal segments in birds of prey as detected by FISH experiments using whole chromosome probes (wcp) derived from chicken (GGA): *Harpia har*pyja (HHA); *Gyps fulvus* (GFU), *G. rueppelli* (GRU); *Gypaetus barbatus* (GBA)*;* Buteoninae (BUT) and *Falco peregrinus* (FPE).

WCP	HHA	GFU/GRU	GBA	BUT*	FPE
**GGA1**	5,6,19,21, 24	7,12,15,19,20,22	7,8p,11,12q	3p/q,6,7,15,18	4, 6
**GGA2**	1,3	2,3,23	1q,2,14q,23q	2,4,20	2,4
**GGA3**	2p,10,18	8,16q,21,24	8q,13,21q,22q	9,13,26,17	6,12
**GGA4**	4,14	1,13	3,16	1,16	1,14
**GGA5**	2q,20	14q,17	15q,20	5,14q	7,10
**GGA6**	8	4q	4q	3q	8
**GGA7**	–	6q	6q	8	1p
**GGA8**	–	10	10	10	10
**GGA9**	–	9q	5q	12	12
**GGA10**	–	18q	9q	19	–

* Including *L albicollis*, *R. magnirostris*, *B. meridionalis* and *A. nitida*.

**Figure 5:**
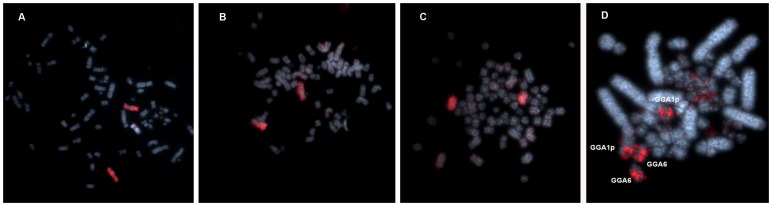
Whole chromosome probe derived from *L.* *albic*ollis which corresponds to a fusion GGA1p/GGA6, confirming this rearrangement as a synapomorphy shared by Buteoninae species. *Asturina nitida* (A), *Rupornis magnirostris* (B) and *Buteogallus meridionalis* (C). In (D), an experiment using the same probe onto *Gallus gallus* metaphase chromosomes.

The availability of whole chromosome probes derived from a Buteoninae (*Leucopternis albicollis*) allowed reciprocal cross-species chromosome painting with *Gallus gallus*, and confirmed that the karyotypes of birds of prey are reshuffled due to the fragmentation of some macrochromosome pairs and fusions of these fragments with microchromosomes [17]. Moreover, the use of both sets of probes (LAL and GGA) in our experiments revealed the existence of a common cytogenetic signature for the Buteonine species analyzed by FISH, namely the association GGA1p/GGA6. This association was confirmed with the use of probe LAL 3, which hybridized onto one submetacentric pair in the three species analyzed herein, while in *Gallus gallus* it hybridized onto the short arm of GGA1 and the whole of GGA6 (figure 5a–d). This supports the close phylogenetic relationship of South-American Buteoninae species, considering that this association has not been observed in other Accipitridae groups so far [18]–[19]. In addition, the apparent similarity found in the species analyzed in [16], from North America, and not included in this study, led us to extend the results to other *Buteo* species. Hence, the genera *Leucopternis*, *Buteogallus*, *Asturina, Rupornis* and *Bute*o may have a close relationship, probably sharing the association GGA1p/GGA6, not found, for example, in the harpy eagle, sometimes included in the same subfamily.

In conclusion, the data obtained in the present study revealed an important cytotaxonomic marker for Buteoninae, showing an exclusive chromosomal synapomorphy, and also raise important issues concerning centromeric distribution and activation. Overall, the findings support the view that Buteoninae should be treated as a monophyletic group inside Accipitridae, and includes not only *Buteo*, but also the so called sub-buteoninae hawks (*Asturina, Rupornis* and *Leucopternis*). The analysis of Holarctic species of Buteoninae could clarify the relationship among the species of this taxon.
